# Selective Induction of DNA Repair Pathways in Human B Cells Activated by CD4^+^ T Cells

**DOI:** 10.1371/journal.pone.0015549

**Published:** 2010-12-16

**Authors:** Xiaosheng Wu, Renee C. Tschumper, Albert Gutierrez, Stephen A. Mihalcik, Grzegorz S. Nowakowski, Diane F. Jelinek

**Affiliations:** 1 Department of Immunology, College of Medicine, Mayo Clinic, Rochester, Minnesota, United States of America; 2 Department of Internal Medicine, College of Medicine, Mayo Clinic, Rochester, Minnesota, United States of America; National Institute on Aging, United States of America

## Abstract

Greater than 75% of all hematologic malignancies derive from germinal center (GC) or post-GC B cells, suggesting that the GC reaction predisposes B cells to tumorigenesis. Because GC B cells acquire expression of the highly mutagenic enzyme activation-induced cytidine deaminase (AID), GC B cells may require additional DNA repair capacity. The goal of this study was to investigate whether normal human B cells acquire enhanced expression of DNA repair factors upon AID induction. We first demonstrated that several DNA mismatch repair, homologous recombination, base excision repair, and ATR signaling genes were overexpressed in GC B cells relative to naïve and memory B cells, reflecting activation of a process we have termed somatic hyperrepair (SHR). Using an *in vitro* system, we next characterized activation signals required to induce AID expression and SHR. Although AID expression was induced by a variety of polyclonal activators, SHR induction strictly required signals provided by contact with activated CD4^+^ T cells, and B cells activated in this manner displayed reduced levels of DNA damage-induced apoptosis. We further show the induction of SHR is independent of AID expression, as GC B cells from AID -/- mice retained heightened expression of SHR proteins. In consideration of the critical role that CD4^+^ T cells play in inducing the SHR process, our data suggest a novel role for CD4^+^ T cells in the tumor suppression of GC/post-GC B cells.

## Introduction

Among all types of hematologic malignancies, more than 75% of patients in the United States are classified as having non-Hodgkin's lymphomas, Hodgkin's disease, chronic lymphocytic leukemia, or multiple myeloma [1], [2]. Of note, each of these derive from germinal center (GC) or post-GC B cells, thereby raising the important question of what makes mature B cells so uniquely predisposed to malignant transformation. The likely answer is that the GC reaction itself renders B cells highly susceptible to acquisition of non-immunoglobulin mutations and genomic instabilities [3], [4], [5], [6], [7], and therefore functions as the “bottleneck” of the genetic wellness of B lineage cells. Consistent with this notion, various cytogenetic abnormalities are notoriously associated with this group of malignancies. However, it remains unclear how the mutations and/or genomic instabilities that are the inevitable by-product of the genome-altering process of somatic hypermutation are suppressed during normal GC reactions, and how this tumor suppression mechanism fails in B lineage malignancies. To better understand these questions, it is essential to study in greater depth the mechanisms governing DNA repair in GC B cells.

In all somatic cells, there is a delicate balance between ongoing levels of DNA damage and repair activity mediated by constitutively expressed DNA repair proteins. The consequences of upsetting this balance by increasing the level of DNA damage or by mutational inactivation of DNA repair genes are highly deleterious and result in the development of cancers in humans and in mouse models [8], [9], [10], [11]. Furthermore, many human sporadic cancers also possess hallmarks of DNA repair deficiencies such as cytogenetic abnormalities, microsatellite instability (MSI), and resistance to DNA damaging therapies [12]. GC B cells have an added burden to contend with, i.e., collateral DNA damage induced by the highly mutagenic enzyme, AID. AID is necessary for the physiological somatic hypermutation (SHM) and class switch recombination (CSR) of immunoglobulin (Ig) genes, and it is now known that AID also causes pathogenic off-target mutations to many other genomic loci [5], [13] and results in tumor development [14], [15], [16] and progression [17]. The additional burden of AID's mutagenic activity raises the tantalizing possibility that B lineage cells require significant additional repair capacity supplementary to constitutively expressed DNA repair factors in order to maintain the tumor suppression balance. We hypothesize that such additional DNA repair capacity results from the temporal induction of expression of various DNA repair genes specifically in GC B cells, and we term this putative tumor suppressive DNA repair mechanism somatic hyperrepair (SHR). In this study, we demonstrate the existence, composition, and function of SHR in GC B cells and discuss its possible role in the development of certain hematologic malignancies.

## Materials and Methods

### Ethics statement

Mayo Clinic Institutional Review Board approval was obtained for use of human blood and tonsil tissue. Informed consent was not required as this material is considered by the Institutional Review Board as waste material generated during either blood donation or surgery. In addition, patient samples arrive de-identified in the laboratory. Institutional Animal Care and Use Committee (IACUC) approval (Mayo Clinic IACUC protocol number A14207) was obtained for studies involving B lymphocytes isolated from mice. Mice were maintained under clean housing conditions at all times with no more than 5 mice per cage. Mice were euthanized under conditions of gradually increasing concentrations of CO_2_ according to Mayo Clinic IACUC guidelines.

### Cells

Mononuclear cells were isolated using Ficoll-Hypaque gradient centrifugation. Human B cells were enriched to >98% purity by magnetic cell separation using a Human B Cell Enrichment kit (StemCell Technologies, Vancouver, British Columbia, Canada). Human B cell subsets were then further purified into naïve (IgD^+^/CD38^−^/CD27^−^), GC (CD19^+^CD38^+^) and memory (IgD^−^/CD38^−^/CD27^+^) populations by FACS sorting on a FACSAria Cytometer (BD, San Jose, CA). Tonsillar GC B cell subset centroblasts (CB) and centrocytes (CC) used in realtime PCR assays were purified as previously described [18]. Autologous CD4^+^ T cells used for B/T cell coculture were purified from blood mononuclear cells using a human CD4^+^ T Cell Enrichment kit (StemCell Technologies). As a source of primary naïve and memory B cells, we used C57BL/6 wildtype or AID^−/−^ (kindly provided by Dr. T. Honjo) mice. Following euthanasia as described above, murine naïve (B220^+^/GL7^−^) and GC (B220^+^/GL7^+^) B cells were purified by FACS sorting from Peyer's patch mononuclear cells isolated from wildtype or AID^−/−^ mice. All antibodies used in FACS analysis and sorting were purchased from BD Biosciences (San Jose, CA).

### B cell *in vitro* activation conditions

Three-day *in vitro* activation of human B cells was carried out essentially as described [19]. Briefly, purified B cells were stimulated for 3 days with 2.5 µg/ml of CpG (oligodeoxynucleotide 2006, 5′-TCGTCGTTTTGTCGTTTTGTCGTT, synthesized by an in-house core facility), 0.5 µg/ml shrCD40L/TNF-related activation protein (Fitzgerald Industries International, Acton, MA), 2 µg/ml of polyclonal anti-Ig (agonistic anti-IgA, IgG, IgM F(ab')_2_ Abs; Jackson ImmunoResearch Laboratories, Inc., West Grove, PA); anti-Ig/CD40 ligand (2 µg/ml agonistic anti-IgA, IgG, IgM F(ab')_2_ Abs and 0.5 µg/ml shrCD40L/TNF-related activation protein) in the presence of IL2 (20 U/ml; PeproTech, Rocky Hill, NJ) and IL10 (50 ng/ml; PeproTech). For the activation of B cells through co-culturing with activated T cells, 6-well microtiter plates were coated with 10 µg/ml anti-human CD3 (clone UCHT1, R&D Systems, Minneapolis, MN) and 10 µg/ml anti-human CD28 (BD Biosciences) in PBS at 4°C overnight. Two million purified B cells and 4×10^6^ autologous CD4^+^ T cells were co-cultured in anti-CD3/anti-CD28 coated wells for 3 days in the presence of 20 U/ml IL2 and 50 ng/ml IL10. Activated B cells and T cells were re-purified upon harvest using B cell and T cell enrichment kits, respectively. For B and T cell coculture in transwells, 4×10^6^ purified CD4 T cells were pre-seeded into anti-CD3/anti-CD28 coated wells for 2 hours before adding purified B cells into the upper transwell (pore size of 0.4 µm, Corning Inc., Corning, NY) compartment.

### DNA damage-induced cell apoptosis

Purified peripheral blood B cells were activated *in vitro* with either 2.5 µg/ml CpG or by co-culturing with anti-CD3/CD28 activated CD4^+^ T cells for 2 days in the presence of 20 U/ml of IL2 and 50 ng/ml of IL10. The DNA damaging agents doxorubicin or cisplatin were added to activated B cells to reach the final concentration of 0.5 µM and 20 µM, respectively. After an additional 24 hours in culture, B cell apoptosis was assessed by FACS analysis using annexin-FITC (Invitrogen, Carlsbad, CA)/propidium iodide/CD19-allophycocyanin staining.

### Immunohistochemistry (IHC) staining

Conventional IHC staining on paraffin-embedded tonsillar tissue sections was carried out using an automatic processor and monoclonal anti-MLH1, anti-MSH2 and anti-MSH6 primary antibodies from Biocare Medical (Concord, CA); primary anti-AID antibody from Cell Signaling (Danvers, MA); and HRP-conjugated secondary antibody (Biocare). Stained sections were then developed with Betazoid DAB as a chromogen (Biocare).

### Western blotting

Protein extracts from B cell subsets or chromatin pellets were analyzed by Western blotting as described elsewhere [20]. Probing antibodies against human β-actin, MSH2, MSH6, MLH1, PMS2, UNG, RAD51, ATM, MRE11, RAD50, NBS1, Ku70, and Ku80 were from Santa Cruz Biotechnology (Santa Cruz, CA). Anti-RPA1 and anti-CHK1 antibodies were purchased from R&D Systems (Minneapolis, MN). Anti-ATR and anti-BRCA1 were kindly provided by Dr. Zhenkun Lou (Mayo Clinic).

### Real-time RT-PCR

One microgram of total RNA isolated using TRIzol reagent (Invitrogen) was used for reverse transcription using the First Strand cDNA kit from GE Healthcare (Piscataway, NJ). The resulting cDNA was diluted into a final volume of 200 µl, of which 5 µl was then used for a 20 µl PCR reaction with 4 pmols of primers. DNA repair primers and RT^2^ SYBR®Green master mix were purchased from SABiosciences (Frederick, MD) while common primers for both mouse and human AID were synthesized in-house (hmAID-F: CCAWTTCAAAAATGTCCGCTGGGC; hmAID-R: AGGAGGTGAACCAGGTGACGCG). Real-time PCR was run in a 384-well plate on an ABI Prism 7900HT RealTime System (Applied Biosystems, Foster City, CA).

### Chromatin fractionation assay

Chromatin fractionations were carried out essentially as described previously [21] using 4×10^6^ purified B cells.

## Results

### Expression of select DNA repair pathway proteins is induced in GC B cells

**Figure 1:**
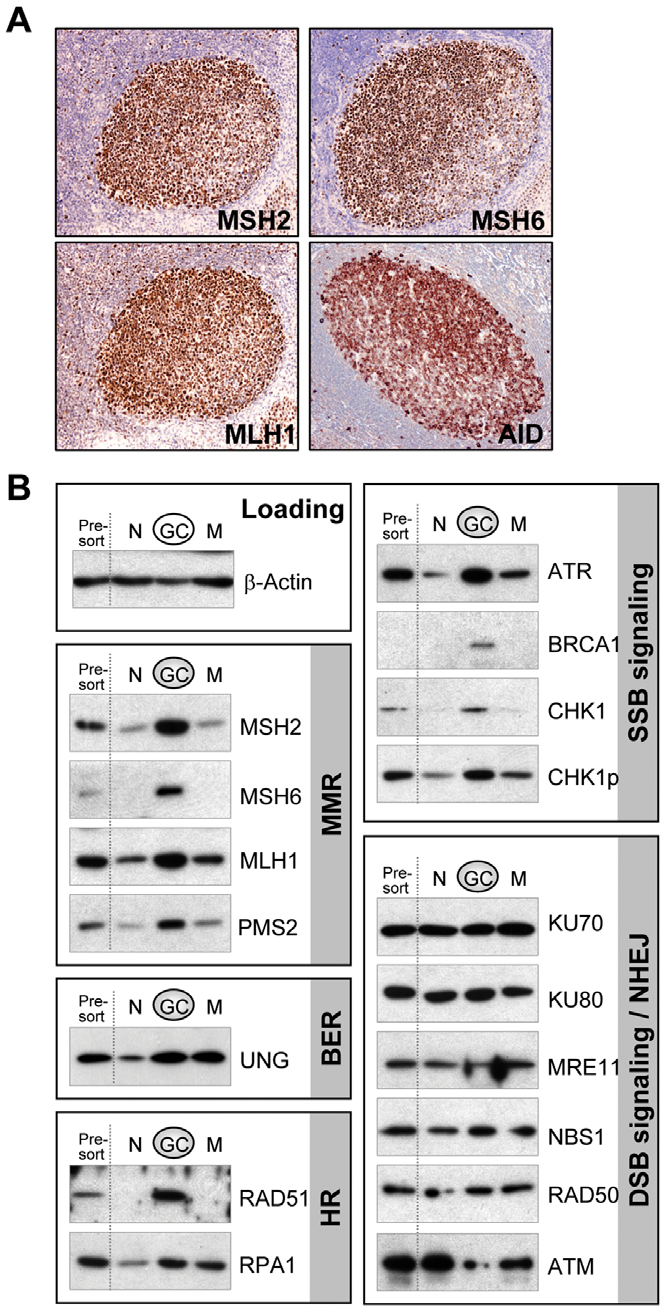
DNA repair proteins are selectively overexpressed in GC B cells. **A.** IHC staining of tonsillar tissue sections with antibodies against DNA mismatch repair proteins MSH2, MSH6, MLH1, and AID. **B.** Western detection of various DNA repair proteins in total tonsillar B cells (presort), naïve (N), GC, and memory (M) B cells. The data are representative of three independent experiments.

Gene targeting studies in mice have shown that DNA mismatch repair (MMR) genes are indispensable for SHM, CSR, and lymphoma suppression of B cells [9], [22], [23], and that even MMR haploinsufficient mice exhibit subtle phenotypes [9], [23]. These observations reveal that MMR plays a critical role within B cells and further suggests that MMR functions in a dose-dependent manner. We therefore hypothesized that the expression levels of MMR components and other DNA repair pathway proteins would increase in GC B cells and constitute the postulated SHR apparatus. Therefore, we first examined the expression levels of MMR proteins in GCs of human tonsillar tissue sections by IHC. Remarkably, we found that all MMR proteins tested (MSH2, MSH6, and MLH1) were highly expressed in the majority of cells localized within the tonsillar GC compartment (Figure 1A) and the pattern of expression was similar to that of AID, a known GC B cell specific protein. Furthermore, MMR proteins were expressed within the nucleus (data not shown), a pattern of expression that is similar to constitutively expressed MMR proteins [20]. These data demonstrate that MMR factors are indeed robustly induced and properly localized in a group of GC cells.

To determine if the cells overexpressing MMR proteins were indeed GC B cells and to determine if other DNA repair factors were also induced in these cells, we next systematically evaluated the expression of key DNA repair proteins of various pathways in purified tonsillar naïve, GC, and memory B cell subsets. As expected, the MMR proteins MSH2, MSH6, MLH1, and PMS2 were indeed highly expressed in purified GC B cells compared to expression levels in naïve B cells (Fig. 1B), suggesting that GC B cells have been induced to express significantly elevated levels of MMR proteins. Moreover, we observed that many other DNA repair proteins were also induced similarly in GC B cells, including the base excision repair (BER) factor UNG, homologous recombination (HR) protein RAD51, single strand DNA binding protein RPA1, and single strand DNA break signaling molecules ATR, BRCA1, and CHK1. Furthermore, expression levels of these DNA repair proteins return to naïve B cell levels in memory B cells, with the exception of UNG and RPA1 proteins that remain moderately expressed, suggesting the induction of these DNA repair proteins is GC B cell specific. Strikingly, key components of the non-homologous end joining (NHEJ) pathway, Ku70 and Ku80, and factors of the double strand DNA break (DSB) signaling pathway MRE11, NBS1, RAD50, and ATM, were not detectably induced in GC B cells, but instead were abundantly expressed in a constitutive manner in all B cell subsets. Our results suggest that the select DNA repair systems, MMR, HR, BER and the ATR signaling pathway, collectively constitute the SHR apparatus in GC B cells.

**Figure 2:**
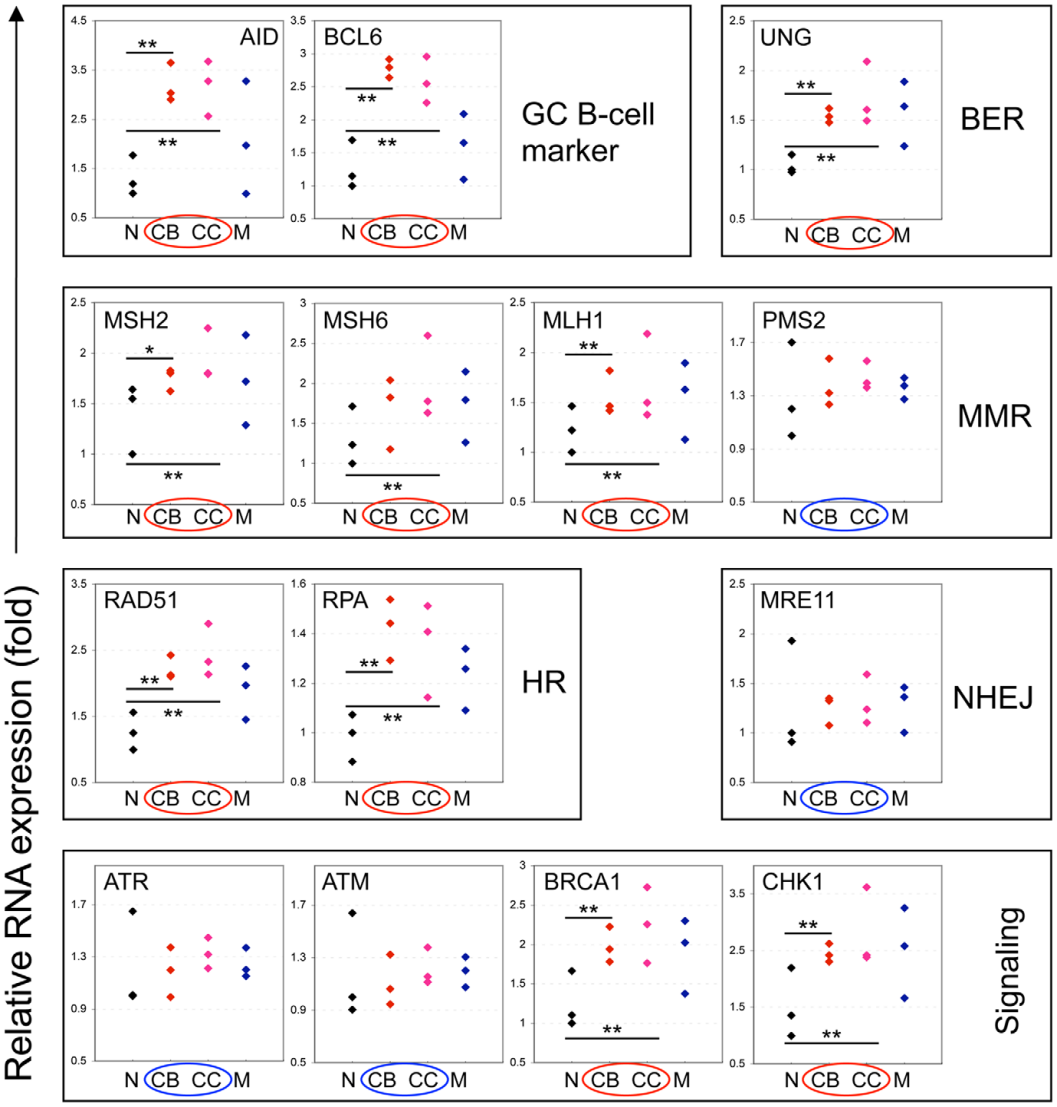
Real-time RT-PCR quantification of mRNA of SHR repair genes in tonsillar naïve (N), GC centroblast (CB), GC centrocyte (CC) and memory (M) B cells. Three tonsil pools each prepared from 6 individual tonsils were used. Genes induced transcriptionally in GC B cells are marked with the red circles while the ones not induced are highlighted with the blue circles. Statistical significance: *p<0.05, **p<0.01.

To determine if the induction of SHR factors occurs at the transcriptional level in GC B cells, we used real-time RT-PCR to quantify the relative expression of mRNA of the repair genes of interest in purified tonsillar naïve, GC, and memory B cells. With the exception of PMS2 and ATR, we found that the mRNA levels of SHR factors were in full accordance with their protein expression (Fig. 2) suggesting the induction occurred at the transcriptional level. With respect to PMS2, it is known that its expression is regulated at the protein level [24]. Consistent with protein expression levels, the NHEJ repair and DSB checkpoint components were not induced at the mRNA level either. Our data are also in agreement with previous microarray studies [18], [25] showing that some DNA repair factors along with other genes are overexpressed in GC B cells. Taken together, we have demonstrated that components of the MMR, HR, and BER pathways and ATR DNA damage checkpoint are selectively induced in GC B cells, collectively constituting the GC-specific SHR machinery.

### SHR proteins are engaged in DNA repair resulting in higher DNA repair capacity

**Figure 3:**
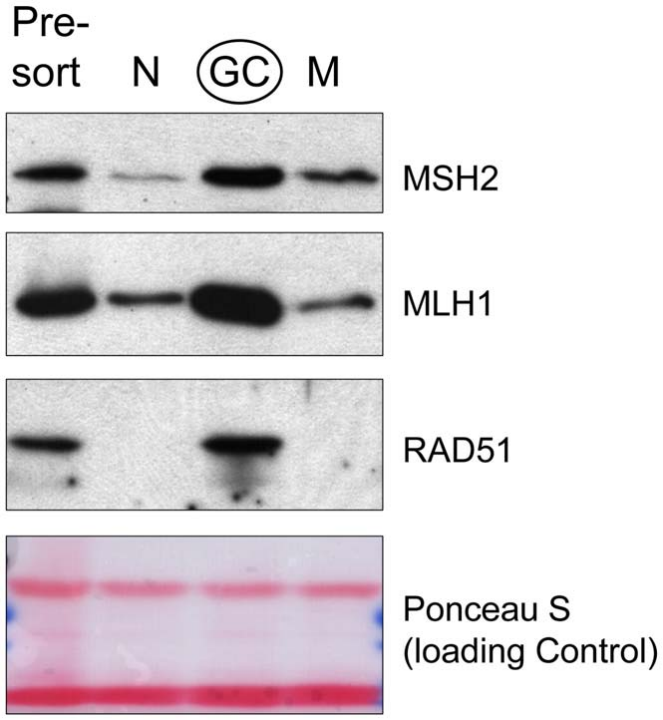
Induced SHR proteins in GC B cells are associated with chromatin, indicative of functional engagement in DNA repair *in vivo*. Western blot detection of various DNA repair proteins in insoluble chromatin fractions of total tonsillar (presort), naïve (N), GC, and memory (M) B cells.

We next asked if the induction of SHR proteins is associated with their DNA repair functions in GC B cells *in vivo*. The total cellular pool of SHR proteins, like other DNA repair proteins, is likely to exist in a soluble form as well as an insoluble form (chromatin-bound). Regarding the latter, it has been suggested that the level of chromatin-bound DNA repair proteins is a steady-state representation of endogenous ongoing DNA repair activity [26], [27]. We took advantage of this knowledge to address this question, and we first determined the levels of MSH2, MLH1, and RAD51 proteins associated with chromatin. As presented in Figure 3, after stringent extraction and washing, significantly higher levels of MSH2, MLH1, and RAD51 remained in the chromatin-bound fraction in GC B cells as compared with naïve and memory B cells. These results suggest that the newly induced levels of SHR factors are actively participating in GC B cell DNA repair. At the same time, we also performed *in vitro* DNA mismatch repair activity assay on the soluble fractions, and we found that GC B cells exhibited a higher level of DNA mismatch repair activity than did similar fractions isolated from naïve and memory B cells. Thus, we observed on average, a 3.5-fold higher level of activity in GC B cells; however, this was somewhat variable between experiments (data not shown). Taken together, our data are compatible with the notion that increased expression of SHR proteins does result in a heightened level of DNA repair activity in GC B cells.

### Induction of SHR and induction of AID are independent processes

**Figure 4:**
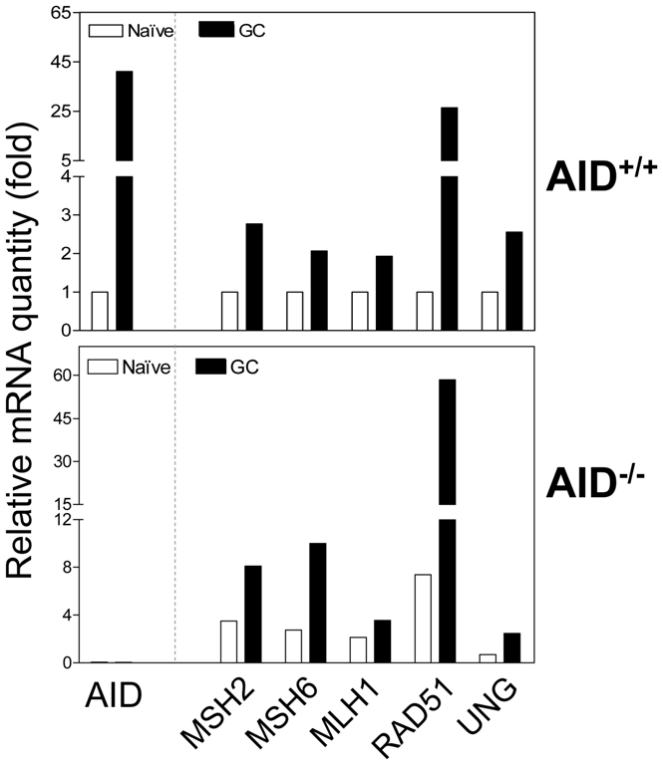
Induction of SHR is AID-independent. Peyer's patch naïve (B220^+^/GL7^−^) or GC (B220^+^/GL7^+^) B cells from AID^+/+^ or AID^−/−^ mice were FACS-sorted, and mRNA levels were then quantitated using real-time RT-PCR. The data are representative of two independent experiments each using Peyer's patches pooled from 6 mice.

Because AID imposes a great genotoxic stress on GC B cells, we next questioned if the induction of SHR factors was simply a stress response to AID-mediated DNA lesions. To that end, we quantitated mRNAs of various DNA repair factors and AID in naïve and GC subsets isolated from wildtype or AID^−/−^ mice. In wildtype mice, GC B cells expectedly expressed about 40-fold more AID, 30-fold more RAD51, and 2-3 fold higher levels of MSH2, MSH6, MLH1, and UNG transcripts compared with naïve B cells. In AID^−/−^ mice, however, GC B cells displayed similar induction levels of the SHR factors MSH2, MSH6, MLH1, RAD51, and UNG as compared to naïve B cells (Fig. 4), suggesting that the programmed SHR induction is neither due to the stress response of AID-mediated DNA lesions nor due to any other AID-mediated mechanisms. Interestingly, we also noticed that the absolute mRNA levels of those repair factors are higher in both naïve and GC B cells of AID^−/−^ mice compared to that of wildtype mice. This may reflect ongoing hyperplasia reportedly occurring in lymphoid follicles of AID^−/−^ mice [28].

### SHR is specifically induced through CD4^+^ T cell-dependent B cell activation

**Figure 5:**
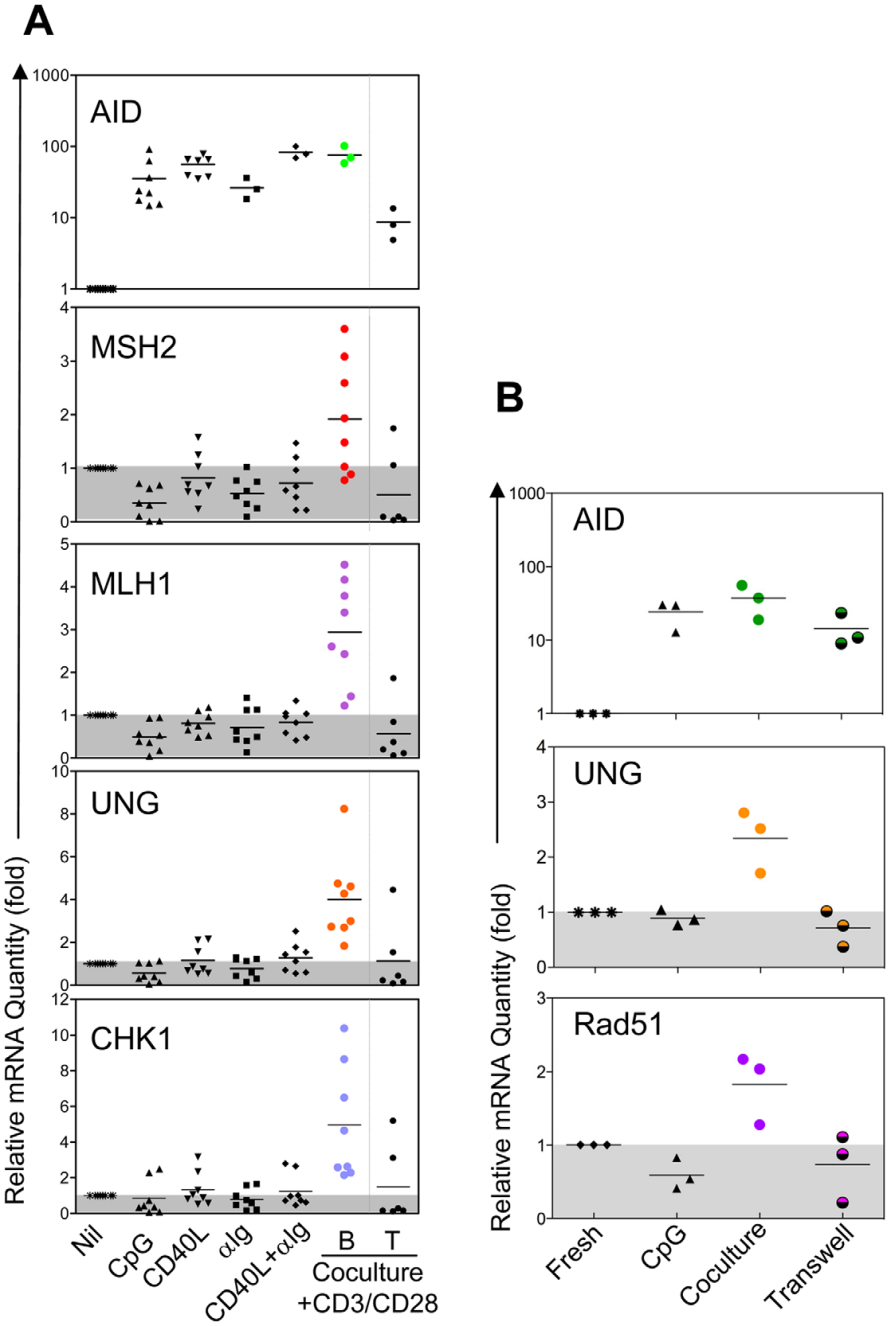
*Ex vivo* induction of SHR and AID. **A.** Purified peripheral blood B cells were stimulated with CpG, CD40 ligand, and/or anti-Ig, in the presence of IL2 and IL10 for 3 days, or coculture with anti-CD3 and anti-CD28 activated CD4 T cells for 3 days. **B.** B cells were activated by anti-CD3 and anti-CD28 activated CD4 T cells through direct coculture, transwell coculture, or activated with CpG for 3 days. Expression of select representative SHR genes were analyzed in activated B cells and activated T cells as a control (B cells and T cells were repurified when T/B direct coculture was the mode of activation) by real-time RT-PCR.

Several studies have shown that various B cell stimuli can induce AID expression *in vitro* prompting us to ask what signaling pathway(s) leads to the induction of SHR. To address this question, we activated pure peripheral blood B cells *in vitro* with various stimuli, attempting to reconstitute at least in part B cell activation events of the GC. As shown in Fig. 5A, AID expression was readily induced by all stimuli including co-culture with activated CD4^+^ T cells. Surprisingly, significant induction of SHR factors was only observed when resting B cells were activated by co-culture with activated CD4^+^ T cells, while stimulation with CpG, CD40L, anti-Ig, or CD40L/anti-Ig had little effect on the expression of SHR factors. Such induction could not come from the residual contaminating T cells since those DNA repair genes are not induced in activated T cells (Fig. 5A, far right column). Of note, although post-cocultured T cells did show some AID activity, we attribute this to the presence of a small number of contaminating B cells as the result of imperfect B/T cell separation following coculture. In addition, when CD8^+^ T cells were used instead for the coculture, SHR induction in B cells was not detected (data not shown). This observation suggests that AID and SHR are each activated through distinct GC-specific signaling pathways, and that unique signals from CD4^+^ T cells are necessary to activate SHR in B cells. However, co-culturing B and T cells in transwells was not sufficient to activate the induction of SHR suggesting that physical contact between B and T cells is necessary (Fig. 5B). Among the B cell stimuli employed, CpG stimulation induced the most robust level of B cell proliferation, yet SHR was not induced under these conditions. This suggests that SHR is not induced simply as a function of cell proliferation. Overall, these results suggest that effective tumor suppression of post-GC B cells may depend on specific signals delivered by CD4^+^ T cells.

### SHR protects B cells from DNA damage-induced apoptosis

**Figure 6:**
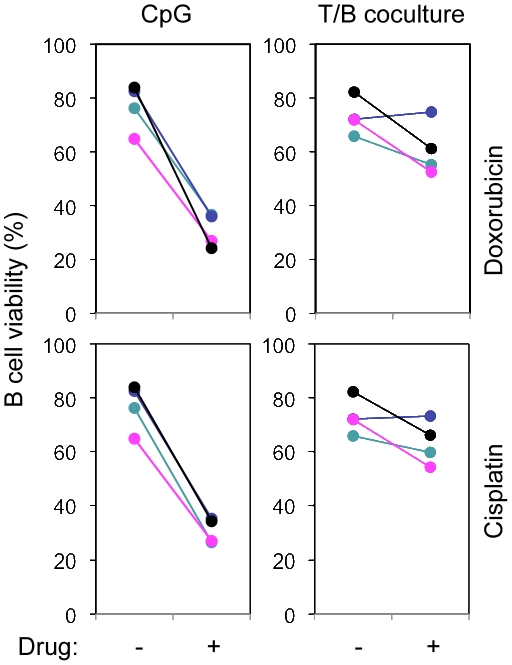
SHR functions in protecting B cells from DNA damage-induced cell apoptosis. Two day-activated peripheral blood B cells with CpG or coculture with anti-CD3 and anti-CD28 activated T cells were further treated with 0.5 µM doxorubicin or 20 µm cisplatin. B cell apoptosis was analyzed using Annexin-FITC/PI/CD19-APC staining. Data include 4 independent experiments.

Ideally, DNA damage is faithfully repaired. However, it is also possible that repair is imprecise and results in oncogenic mutations and ultimately transformation, or cell apoptosis if the damage is irreparable. We hypothesized that SHR would mitigate or attenuate both of the latter events. To address this hypothesis, we first asked whether induction of SHR would protect B cells from DNA damage-induced apoptosis. To that end, we activated purified peripheral blood B cells *in vitro* and then challenged the activated B cells with DNA-damaging agents doxorubicin or cisplatin. As shown in Fig. 6, when B cells were activated with CpG, a stimulus shown to lack the ability to induce SHR (Fig. 5), cells were very sensitive to the treatment with either doxorubicin or cisplatin. In contrast, when B cells were activated by co-culture with activated CD4^+^ T cells, a stimulus that induces the expression of SHR proteins, B cell sensitivity to doxorubicin- and cisplatin-induced apoptosis was significantly reduced. These data further complement our earlier observations suggesting that SHR induction results in heightened DNA repair capacity in GC B cells.

## Discussion

Mounting evidence has shown that expression of AID in GC B cells poses a serious threat to genomic integrity and may induce oncogenesis unless there is additional DNA repair capacity. Here we show that GC B cells are indeed induced by specific signals to express increased levels of various DNA repair pathway factors. We postulate this occurs specifically to counteract the mutational threat posed by AID, a mechanism we have termed as SHR. We further demonstrated that SHR collectively consists of MMR, BER, HR and ATR signaling pathways. It is important to note that elevated expression of a single DNA repair protein is unlikely to result in enhanced DNA repair activity unless this protein plays a rate-limiting role in the repair pathway. However, our data demonstrating that a panel of pathway-wide factors is simultaneously induced suggests that this response likely does result in higher repair activities. Indeed, our preliminary DNA repair activity data taken together with data obtained from our DNA damage protection assays provide support for the notion that SHR is functionally significant to GC B cells. Moreover, our data are consistent with a previous observation that DNA repair is not deficient in GC B cells [29] and we further extend this work by showing that GC B cell repair capacity is not merely equivalent to naïve and memory B cells, but instead is significantly increased.

In addition to increases in DNA repair capacity, increases in DNA repair fidelity are also necessary to suppress the accumulation of mutations. The SHR machinery achieves these twin goals by the selective inclusion of high fidelity DNA repair pathways. While both NHEJ/ATM and HR/ATR axes can repair blunt end DSBs [30], SHR induces the error-free HR/ATR repair axis rather than the error-prone NHEJ/ATM pathway. SHR's selective inclusion of high fidelity DNA repair proteins is entirely consistent with its proposed mutation avoidance role in tumor suppression of post-GC B cells.

It is known that expression of DNA repair genes can be regulated by cell cycle regulators and that GC B cells are undergoing extensive cell proliferation. However, it is unlikely that SHR induction resulted from cell cycle effects in these experiments, since the other B cell stimulators employed in this study, i.e., CpG, CD40L, and anti-Ig, induced robust B cell proliferation yet still did not induce SHR expression. Furthermore, the selective nature of the DNA repair pathway induction in GC B cells suggests a mechanism with more specificity than the global changes accompanying proliferation. It would be interesting to characterize the cell proliferation profile and cell cycle characteristics of the SHR-expressing B cell subset, however, this will require the use of monoclonal B cells from mice or use of human B cell lines which may not be representative of normal GC B cells. We recognize that some of the SHR genes remain moderately expressed in newly emerged tonsillar memory B cells, though they are clearly downregulated in more mature subsets. This intermediate expression may reflect a real physiologic kinetic phenomenon in which CD27 induction on memory B cells and extinction of SHR are somewhat temporally staggered or may simply reflect the imperfect separation between GC and memory B cells during cell isolation. In either case, the clear distinction between GC and mature memory B cells implicate SHR as a GC-specific event.

Our data indicate that ATR is induced at the protein level but not at the transcriptional level while the other ATR pathway proteins, CHK1 and BRCA1, are induced at both levels. Recently, Melnick and colleagues described a feedback loop in which the transcriptional repressor BCL6 represses the expression of ATR and CHK1 in GC B cells [31], [32]. These findings do not contradict our data since it remains possible that ATR and CHK1 are expressed at induced levels in GC B cells relative to expression levels in naïve and memory B cells despite coincident BCL6 repression. Furthermore, the authors made another important observation that BCL6-mediated ATR repression is rather brief and is readily overridden by CD40 signaling, the major co-stimulatory signaling pathway triggered by CD4^+^ T cell engagement. Consistent with this notion and with our results, microarray analysis showed that CHK1 expression levels were indeed induced in GC B cells as compared with naïve and memory B cells [18].

Under normal circumstances, B cell encounters with CD4^+^ T helper cells in the GC results in induction of SHR as well as the expression of AID. However, our data show that many other B cell stimuli can activate the expression of AID without the induction of SHR. The independence of AID and SHR raises the possibility that stimuli resulting in AID expression unbalanced by SHR may ultimately lead to DNA repair deficits that may result in accumulation of mutations. Specifically, this observation predicts that physiologic circumstances like the opportunistic ligation of CD40 receptors on B cells by soluble CD40L, the activation of B cells by CpG, and the activation of B cells through the B cell receptor by T cell independent antigens may thus be more likely to result in the oncogenic transformation of B cells. Consistent with this notion, mice transgenic for CD40L, or CD40L functional equivalent latent membrane protein 1 (LMP1) of Epstein-Barr virus do develop various B cell malignancies [33], [34]. Similarly, *in vitro* experiments have shown that CpG stimulation could readily induce non-clonal cytogenetic abnormalities suggesting newly acquired genomic instabilities (Wu et al., submitted for publication).

It is readily accepted that increased EBV infection in B cells and weakened immune surveillance play a major role in the increased incidence of B cell lymphoma in HIV^+^ patients. However, it is difficult to fully explain how EBV negative lymphomas arise in this population and why perforin knockout mice defective in immune surveillance only showed a moderate increase of spontaneous B cell lymphoma at late onset [35]. Our current data suggest a possible novel function for CD4^+^ T cells in suppressing B cell tumorigenesis, providing a rational complementary and/or alternative explanation for this lingering paradox. Moreover, our data also suggest that CD4^+^ T cells act as a safeguard to prevent nonspecific B cell activation leading to imbalanced AID expression.

With respect to this latter point, it is interesting that some HIV-related lymphomas exhibit microsatellite instability (MSI) despite lacking any apparent deficiency of MMR proteins [36]. This discrepancy suggests that there was a transient MMR deficiency at some point in the history (likely in GCs) of the malignant B cell. Our data suggesting that SHR induction is necessary to mitigate DNA damage in GC B cells such as MSI is consistent with these observations and offers a novel interpretation that MSI in these lymphomas was likely acquired during the GC reaction due to compromised SHR induction rather than from constitutive defects in MMR. In this regard, gene targeting experiments in mice have shown that mutations at A/T positions in the neighborhood of AID target “C” sites are exclusively introduced by the DNA MMR system during SHM, and that compromised MMR leads to the reduction of mutation frequencies at A/T positions in Ig genes [37]. Thus, we hypothesize that if defective SHR induction could lead to the development of B cell malignancy, those malignant cells would harbor skewed A/T mutation frequencies in their Ig genes. One example of such a scenario could be lymphomas that develop in HIV-infected patients whose SHR-inducing CD4^+^ T cells are severely suppressed. To that end, in preliminary studies, we analyzed the A/T mutation frequencies of Ig sequences from 17 EBV-negative HIV-related lymphoma samples and 39 Ig sequences from normal B cells. Our preliminary yet tantalizing data indicate that A/T mutations in HIV related lymphomas are indeed skewed in a manner suggestive of compromised SHR when compared to that of normal B cells (Wu, unpublished data). However, direct involvement of SHR in lymphomagenesis will require further thorough investigation, which is currently under way in our laboratory.

Given our finding that CD4^+^ T cells play an essential role in the induction of SHR in GC B cells, we believe that further identification of the specific receptors and signaling elements underlying induction of SHR will broaden our understanding of the etiology of mature B cell malignancies. Such an understanding will also enable us to target these molecules therapeutically to prevent lymphomagenesis in HIV-infected patients and organ transplant recipients.

## References

[1] Horner MJRL, Krapcho M, Neyman N, Aminou R, Howlader N SEER Cancer Statistics Review, 1975-2006.. http://seer.cancer.gov/csr/1975_2006/.

[2] Morton LM, Wang SS, Devesa SS, Hartge P, Weisenburger DD (2006). Lymphoma incidence patterns by WHO subtype in the United States, 1992-2001.. Blood.

[3] de Yebenes VG, Ramiro AR (2006). Activation-induced deaminase: light and dark sides.. Trends Mol Med.

[4] Liu M, Schatz DG (2009). Balancing AID and DNA repair during somatic hypermutation.. Trends Immunol.

[5] Pasqualucci L, Neumeister P, Goossens T, Nanjangud G, Chaganti RS (2001). Hypermutation of multiple proto-oncogenes in B-cell diffuse large-cell lymphomas.. Nature.

[6] Phan RT, Dalla-Favera R (2004). The BCL6 proto-oncogene suppresses p53 expression in germinal-centre B cells.. Nature.

[7] Wu X, Jelinek DF (2005). A-Miz-ing BCL6.. Nature Immunology.

[8] de Wind N, Dekker M, Berns A, Radman M, te Riele H (1995). Inactivation of the mouse Msh2 gene results in mismatch repair deficiency, methylation tolerance, hyperrecombination, and predisposition to cancer.. Cell.

[9] Edelmann W, Yang K, Umar A, Heyer J, Lau K (1997). Mutation in the mismatch repair gene Msh6 causes cancer susceptibility.. Cell.

[10] Nilsen H, Stamp G, Andersen S, Hrivnak G, Krokan HE (2003). Gene-targeted mice lacking the Ung uracil-DNA glycosylase develop B-cell lymphomas.. Oncogene.

[11] Reitmair AH, Schmits R, Ewel A, Bapat B, Redston M (1995). MSH2 deficient mice are viable and susceptible to lymphoid tumours.. Nat Genet.

[12] Hanahan D, Weinberg RA (2000). The hallmarks of cancer.. Cell.

[13] Liu M, Duke JL, Richter DJ, Vinuesa CG, Goodnow CC (2008). Two levels of protection for the B cell genome during somatic hypermutation.. Nature.

[14] Matsumoto Y, Marusawa H, Kinoshita K, Endo Y, Kou T (2007). Helicobacter pylori infection triggers aberrant expression of activation-induced cytidine deaminase in gastric epithelium.. Nat Med.

[15] Okazaki IM, Hiai H, Kakazu N, Yamada S, Muramatsu M (2003). Constitutive expression of AID leads to tumorigenesis.. J Exp Med.

[16] Perez-Duran P, de Yebenes VG, Ramiro AR (2007). Oncogenic events triggered by AID, the adverse effect of antibody diversification.. Carcinogenesis.

[17] Klemm L, Duy C, Iacobucci I, Kuchen S, von Levetzow G (2009). The B cell mutator AID promotes B lymphoid blast crisis and drug resistance in chronic myeloid leukemia.. Cancer Cell.

[18] Klein U, Tu Y, Stolovitzky GA, Keller JL, Haddad J (2003). Transcriptional analysis of the B cell germinal center reaction.. Proc Natl Acad Sci U S A.

[19] Darce JR, Arendt BK, Wu X, Jelinek DF (2007). Regulated expression of BAFF-binding receptors during human B cell differentiation.. J Immunol.

[20] Wu X, Platt JL, Cascalho M (2003). Dimerization of MLH1 and PMS2 limits nuclear localization of MutLalpha.. Mol Cell Biol.

[21] Mendez J, Stillman B (2000). Chromatin association of human origin recognition complex, cdc6, and minichromosome maintenance proteins during the cell cycle: assembly of prereplication complexes in late mitosis.. Mol Cell Biol.

[22] Li Z, Woo CJ, Iglesias-Ussel MD, Ronai D, Scharff MD (2004). The generation of antibody diversity through somatic hypermutation and class switch recombination.. Genes Dev.

[23] Wei K, Kucherlapati R, Edelmann W (2002). Mouse models for human DNA mismatch-repair gene defects.. Trends Mol Med.

[24] Chang DK, Ricciardiello L, Goel A, Chang CL, Boland CR (2000). Steady-state regulation of the human DNA mismatch repair system.. J Biol Chem.

[25] Longo NS, Lugar PL, Yavuz S, Zhang W, Krijger PH (2009). Analysis of somatic hypermutation in X-linked hyper-IgM syndrome shows specific deficiencies in mutational targeting.. Blood.

[26] Schroering AG, Williams KJ (2008). Rapid induction of chromatin-associated DNA mismatch repair proteins after MNNG treatment.. DNA Repair (Amst).

[27] Zou L, Cortez D, Elledge SJ (2002). Regulation of ATR substrate selection by Rad17-dependent loading of Rad9 complexes onto chromatin.. Genes Dev.

[28] Fagarasan S, Muramatsu M, Suzuki K, Nagaoka H, Hiai H (2002). Critical roles of activation-induced cytidine deaminase in the homeostasis of gut flora.. Science.

[29] Park K, Kim J, Kim HS, Shin HS (1998). Isolated human germinal center centroblasts have an intact mismatch repair system.. J Immunol.

[30] Shiotani B, Zou L (2009). Single-stranded DNA orchestrates an ATM-to-ATR switch at DNA breaks.. Mol Cell.

[31] Ranuncolo SM, Polo JM, Dierov J, Singer M, Kuo T (2007). Bcl-6 mediates the germinal center B cell phenotype and lymphomagenesis through transcriptional repression of the DNA-damage sensor ATR.. Nat Immunol.

[32] Ranuncolo SM, Polo JM, Melnick A (2008). BCL6 represses CHEK1 and suppresses DNA damage pathways in normal and malignant B-cells.. Blood Cells Mol Dis.

[33] Kulwichit W, Edwards RH, Davenport EM, Baskar JF, Godfrey V (1998). Expression of the Epstein-Barr virus latent membrane protein 1 induces B cell lymphoma in transgenic mice.. Proc Natl Acad Sci U S A.

[34] Uchida J, Yasui T, Takaoka-Shichijo Y, Muraoka M, Kulwichit W (1999). Mimicry of CD40 signals by Epstein-Barr virus LMP1 in B lymphocyte responses.. Science.

[35] Smyth MJ, Thia KY, Street SE, MacGregor D, Godfrey DI (2000). Perforin-mediated cytotoxicity is critical for surveillance of spontaneous lymphoma.. J Exp Med.

[36] Duval A, Raphael M, Brennetot C, Poirel H, Buhard O (2004). The mutator pathway is a feature of immunodeficiency-related lymphomas.. Proc Natl Acad Sci U S A.

[37] Rada C, Di Noia JM, Neuberger MS (2004). Mismatch recognition and uracil excision provide complementary paths to both Ig switching and the A/T-focused phase of somatic mutation.. Mol Cell.

